# Supplementary material for: Are carbon emissions trading and green financial instruments synergistic? -Comprehensive quantitative research based on content analysis
Source: PLoS One. 2024 Mar 7;19(3):e0298601. doi: 10.1371/journal.pone.0298601 (PMC10919601; doi:10.1371/journal.pone.0298601)
Supplement: S1 Data — (ZIP) [file pone.0298601.s001.zip › Data/地级市Z_F工作报告.zip!/║╙─╧/┬σ╤⌠╩╨/└┤╫╘╣1⁄2╓┌║┼í╛┬φ┐╦╩2╛▌═°í┐_2012.docx]

各位代表：

现在，我代表市人民政府向大会作工作报告，请予审议，并请各位政协委员和列席人员提出意见。

一、2011年工作回顾

2011年，在省委、省政府和市委的正确领导下，在市人大、政协的监督支持下，市政府团结带领全市人民，围绕“福民强市”总体目标，锐意进取，务实重干，全力打好项目建设、经济转型、机制转换、城市提升、民生改善、环境创优和国际文化旅游名城建设“六加一”攻坚战，圆满完成了市十三届人大三次会议确定的各项目标任务，实现了“十二五”发展的良好开局。初步测算，全年完成地区生产总值2723亿元，比上年增长12.8%。固定资产投资1855亿元，增长27%。地方财政一般预算收入178.3亿元，增长25.5%。社会消费品零售总额954亿元，增长18%。外贸出口14.5亿美元，增长38.1%。城镇居民人均可支配收入和农民人均纯收入分别达到19756元和6532元，增长12%和15%左右。特别是经过全市人民艰苦不懈的努力，我市顺利通过国家卫生城市复审，荣获全国文明城市、国家森林城市称号。

——项目建设步伐加快，产业结构进一步优化。全年实施千万元以上项目3894个，完成投资1592.2亿元。传统产业升级加速。出台了洛阳市老工业基地振兴规划及一系列促进结构调整的政策措施。中信重工特大型锻造基地、伊电集团25万吨高精度铝板带、725所1万吨钛板带、轴研科技重型精密轴承等项目竣工投产；麦达斯轨道交通铝型材、恩梯恩LYC公司汽车轴承等项目开工建设；洛阳石化1800万吨炼油扩能改造、洛玻集团搬迁改造等项目前期工作扎实推进。战略新兴产业不断壮大。出台了战略新兴产业培育等“三大工程”、“一大政策”。中硅高科光伏产业园75兆瓦单晶硅片等项目竣工投产，中航锂电产业园等项目加紧建设。上海超日400兆瓦光伏垂直一体化、中航光电产业基地、微软技术中心等项目开工建设。风电叶片、风电轴承、兽用疫苗、节能环保装备、锂离子电池等技术达到国际国内领先水平。现代服务业加快发展。实施千万元以上服务业项目136个。开元门生态商务区等十大服务业示范项目加快实施。河南移动客服中心等项目竣工投用。863软件孵化器、恒生科技园、通和农副产品物流园、大张物流园等项目顺利推进。国际文化旅游名城战略策划项目全面完成。隋唐城遗址保护展示，龙门、白马寺景区提升等重点示范项目加快推进。老君山·鸡冠洞旅游区晋级国家 5A 级景区。

——经济转型实现新的突破，发展质量进一步提高。自主创新能力持续提升。新建国际联合实验室2个、河南省院士工作站11个、市级以上企业研发中心49个，河柴重工技术中心被认定为国家级企业技术中心。26家省百高、百强企业以及42家重点制造业企业全部建立研发中心。中钢洛耐研究院、中硅高科分别牵头组建了国家、省产业技术创新战略联盟。专利、发明专利申请量均居全省第二位。78项科研成果通过产学研合作信息平台实现产业化。荣获国家科技进步奖2项、省级科技进步奖28项。产业集聚成效明显。各产业集聚区基础设施建设、招商引资、产业培育力度不断加大，特色主导产业基本形成。17家产业集聚区新开工亿元以上项目占全市的72%，规模以上工业主营业务收入占全市的54%。其中，10家产业集聚区被列为省重点示范产业集聚区，数量居全省第一。碧水蓝天工程扎实推进。大力淘汰落后产能，加快实施节能改造、资源综合利用和循环经济项目。污水处理厂及配套管网建设步伐加快，电力、冶金、建材、化工等企业实现达标排放。全市空气质量优良天数达到316天，出境水质断面综合达标率98%以上。

——新农村建设扎实推进，“三农”基础进一步巩固。结构调整、土地流转、生态旅游成效显著。粮食总产达到46亿斤，连续九年实现丰收。新发展牡丹4.87万亩，花卉苗木、蔬菜、林果、畜禽养殖等特色产业比重达60%以上。完成土地流转90万亩。转移农村劳动力142万人，劳务创收85亿元。休闲旅游农业园区达到796个。农业产业化占农业总产值的比重达到58%。农业综合生产能力进一步提升。完成水库除险加固12座，新增有效灌溉面积6.4万亩。植树造林45.4万亩。农村面貌明显改观。新开工新型农村社区87个，建成50个。新建农村社区服务中心103个，硬化通组通户道路 2061公里 ，解决安全饮水30万人，完成村改居177个。全市95%以上行政村建立了卫生保洁长效机制。

——改革开放不断深化，发展活力进一步增强。企事业单位改革取得重大进展。市属国有企业和事业单位改革改制、县（市、区）企事业单位改革实现大头落地。洛轴集团等3户企业政策性破产加快推进。省定煤矿兼并重组、集体林权制度改革确权发证任务全面完成。文化体制改革工作被评为全国先进。民营经济快速发展。建立了民营经济新闻发布会等制度，民营经济发展氛围进一步浓厚。北玻公司等4家民营企业成功上市。民营经济新上千万元以上项目820个，完成投资850亿元，实现增加值占全市生产总值的比重超过50%。招商引资成效显著。全年签订招商引资项目356个，投资总额增长50%；实际利用外资和市外境内资金分别增长46.8%和32.2%。中移动（洛阳）呼叫中心、浙商工业园、国龙物流园等重大项目落户洛阳。对外贸易实现新突破。预计外贸进出口总额达20.5亿美元，增长33.1%。机电产品、高新技术产品出口额占全市的83%，出口结构继续优化。旅游业发展态势良好。第29届中国洛阳牡丹文化节、小浪底观瀑节、河洛文化旅游节、伏牛山滑雪节圆满成功。全年接待游客6870万人次、其中入境游53万人次，旅游总收入350亿元。

——城市形象不断提升，城镇化进程进一步加快。新区建设势头强劲。伊洛大道、洛白路改造等24项市政基础设施项目建成投用，伊滨区路网基本形成。行政副中心、中央商务区、福民安置小区、职教园区等重点区域，龙门大道提升、高铁大道跨伊河大桥等重大项目建设进展顺利，兰迪新型中空玻璃、轴承科技产业园等项目加快实施，南兆域、伊河生态廊道等城乡一体化试点项目全面启动。城区改造成效显著。新建、改扩建市政道路 37.2公里 。洛阳桥扩建、瀛洲北路二期等工程建成投用，中州路、凯旋路等18条市管道路改造提升工程全面完成。丽春东路打通、道北三路打通等工程进展顺利，九都路高架等项目前期工作加快推进。城中村、城郊村和旧城改造实现重大突破，全年完成改造拆迁面积2000万平方米以上，开工安置房1500万平方米，竣工340万平方米。新增城市绿地125万平方米。城市管理水平明显提升。建立了“110城市应急联动服务平台”，80家职能部门联网办理群众咨询和诉求。启动数字化城市管理系统建设，加大市容巡查督办力度，新建公厕303座、垃圾中转站34座，城市环境卫生状况明显改观。交通辐射带动能力不断增强。连霍高速郑州至洛阳段，洛偃、洛宜快速通道建成通车。连霍高速洛阳至三门峡段、郑卢高速洛阳段、洛栾高速及洛吉、洛伊快速通道加快推进。310国道改线、小浪底专用线改造等项目前期工作进展顺利。建成农村公路 188公里 。洛阳机场旅客吞吐量比上年增长28%。

——民生工程加快实施，社会保障体系进一步完善。省定、市定福民实事全面完成，各级财政用于民生的支出达到198亿元，占财政一般预算支出的66.9%。全民创业成绩喜人。发放小额担保贷款13.7亿元，扶持2.2万人成功创业。新增城镇就业12.9万人，基本实现零就业家庭至少有一人就业。社会保障水平不断提高。在全省率先实现城镇职工基本医疗保险市级统筹，城乡居民社会养老保险制度实现全覆盖。提高城乡居民最低生活保障和农村五保供养标准，基本实现应保尽保。建立物价与最低生活保障联动机制，为困难群众发放临时价格补贴1289万元。新开工保障性住房3.5万套168万平方米，竣工71. 4 万平方米 ，6137户住房困难群众乔迁新居。农村敬老院改造、倒房重建等工作加快推进。完成搬迁扶贫2250户10005人。

——社会事业全面进步，社会管理进一步加强。新建、改扩建学校16所，吸引社会力量举办各类学校、幼儿园、教育机构71所，城乡中小学、幼儿园校舍安全改造工程进展顺利。普通高招再创新高。河南推拿学院和洛阳职业技术学院成功升格。优势医院倍增计划加快实施，河科大一附院新区医院建成开业，第五人民医院新区医院、妇儿中心新区医院等项目加紧推进，开工建设县医院4个，新增床位1020张。全市乡镇卫生院实现药物零差率销售。新型农村合作医疗参合率达99.4%。人口自然增长率控制在5.8‰。文化基础设施建设步伐加快，建成公共文化馆16个、图书馆10个。洛阳博物馆新馆全面对外开放。成功举办河南省第五届农民运动会。扎实开展安全生产及食品药品安全专项整治。稳妥推进警务体制改革创新，深入开展打黑除恶扫痞和校园周边环境治理专项行动，城市区和所有行政村实现技防设施全覆盖。积极排查化解矛盾纠纷，社会大局保持稳定。

——政府自身建设不断加强，行政效能进一步提升。坚持依法行政、阳光操作，主动接受人大及其常委会监督，支持政协参政议政，人大代表议案、建议和政协提案办理满意率均达到99.6%以上。加强政府立法，提请市人大常委会审议法规草案2件，颁布政府规章3件。圆满完成了第七届村民委员会换届工作。深入开展“环境创优年”活动，加强机关效能建设，发展环境进一步优化。全面推进政务公开，面向社会公布了市直各部门的服务承诺。认真清理行政审批事项，实现了审批项目全省最少。政府廉政建设和反腐败工作取得新成效。加强军政军民团结，荣获全国双拥模范城六连冠。与此同时，民族宗教、对台工作、外事侨务、统计、审计、人防、气象、地震、档案、史志、残疾人事业等都取得了新的成绩。

各位代表！过去的一年，我们之所以能够实现又好又快的发展，是省委、省政府和市委科学决策、正确领导的结果，是市人大、政协有效监督、鼎力支持的结果，更是680万洛阳人民团结奋斗、锐意进取的结果。在此，我代表市政府，向全市各条战线上的工人、农民、干部、知识分子和广大建设者，向人大代表、政协委员，向各民主党派、工商联、无党派人士和人民团体，向离退休老领导、老同志，向驻洛部队、武警官兵、公安政法干警，向所有关心支持洛阳发展的社会各界人士，表示崇高的敬意和衷心的感谢！

在肯定成绩的同时，我们还要清醒地看到，当前我市经济社会发展中还存在一些亟待解决的问题：一是产业抗风险能力较弱。工业增长对石化、有色、电力等产业的依赖度较高，去年因市场波动和价格倒挂，直接影响了全市经济运行的质量和效益。二是资源环境约束力增大。我市能源原材料行业比重大，不仅单位产值能耗较高，而且能耗总量较大，增加了节能工作的难度。同时，经济发展、人口增多、消费水平提高对环境提出了新的要求，我们既要偿还历史欠账，又要做好当前的环境治理工作，任务十分艰巨。三是改革改制工作有待深化。部分领域的改革尚未全面启动，个别单位改制不够彻底，职工安置还不到位，仍需进一步规范和完善。四是民生保障与社会管理任务繁重。就业、教育、医疗、住房等问题还没有得到很好解决，部分群众生活还比较困难，安全生产、食品安全、社会治安、信访工作还存在薄弱环节，交通拥堵、物业管理等问题逐渐显露。五是机关作风建设有待加强。一些部门职能转变不到位，行政效率低下，处置突发事件能力较弱；一些干部思想僵化，创新意识不强，运作水平不高。对此，我们一定要高度重视，在今年的工作中采取切实有效措施，逐步加以解决。

二、2012年总体工作要求

今年是实施“十二五”规划的重要一年，也是全面推进中原经济区建设的关键之年。当前我们面临的外部环境更加复杂多变。金融危机的影响短期内难以消除，国际市场需求低迷，国内需求增长难度加大，经济增速下行与物价高位运行矛盾交织，加之区域竞争日趋激烈，给我市经济增长带来了较大压力。同时，我们更要看到，国内经济总体向好的基本面没有改变，我市仍处于加快发展的重要战略机遇期。首先，今年国家继续实施积极的财政政策和稳健的货币政策，加大对“三农”、保障性住房、社会事业等领域的投入，保持社会融资规模合理增长。只要我们顺应宏观政策，多渠道争取资金支持，筛选出符合国家政策导向和资金投向的项目，就一定能够保持足够的投资强度，加强我市的薄弱环节建设。其次，《国务院关于支持河南省加快建设中原经济区的指导意见》明确提出，要支持洛阳加快老工业基地调整改造、建设新材料产业基地和有重要影响力的产业创新中心。我市产业基础雄厚、科技实力突出、人才优势明显，只要我们善于创新机制，推动产学研军深度融合，就一定能够加快产业结构优化升级、打造老工业基地振兴转型品牌。第三，中央、省明确提出要推动文化大发展、大繁荣，加快建设华夏历史文明传承创新区。我市文化底蕴深厚、资源优势独特，只要我们善于挖掘资源，策划并实施一批文物保护、文化资源开发利用项目，凝炼一批特色鲜明的河洛文化符号，就一定能够在增强文化软实力、提升城市竞争力方面走在全省前列。第四，国家明确提出要支持我省建设中原历史文化旅游区、黄河文化旅游带等重点旅游景区和精品旅游线路。我市旅游资源丰富、自然人文景观众多，旅游人数稳居全省首位，只要我们抓住机遇，加大投入，完善服务设施，大力宣传推介，就一定能够提高游客的消费预期，带动本地酒店、餐饮、娱乐、商业等服务业的发展。第五，中央、省新一轮的扶贫攻坚计划开始实施，《中国农村扶贫开发纲要（2011－2020年）》将我市栾川、嵩县、洛宁、汝阳4县列入秦巴片区扶贫规划。只要我们深入研究政策，做好基础工作，就一定能够打赢新一轮扶贫开发攻坚战，实现贫困地区群众脱贫致富。第六，近年来，我市固定资产投资增速较快，一些打基础、增后劲的重点项目陆续投产，产业集聚功能不断提高，“三化”协调发展格局正在形成。特别是去年以来实施的“六加一”攻坚战成效明显，社会大局和谐稳定，干事创业氛围浓厚，为我市今后的发展奠定了坚实的基础。

基于上述分析，今年政府工作的总体要求是：以科学发展为主题，以加快发展方式转变为主线，紧紧抓住并切实用好中原经济区建设的重大机遇，增强忧患、化危为机，积极作为、持续求进，提振信心、求实求效，以“当好一个示范、打造两大品牌”为战略定位，以持续和提升“六加一”攻坚战为重中之重，确保我市经济、社会、生态建设主要指标和协调发展程度高于全省平均水平，城乡居民收入的增长、幸福指数的提升高于全省平均水平，确保社会大局稳定。

初步确定2012年全市经济社会发展的预期目标是：地区生产总值增长11%左右，公共财政预算收入增长15%左右，固定资产投资增长25%以上，社会消费品零售总额增长17%左右，外贸出口增长25%以上，实际利用外资增长25%以上，城镇居民人均可支配收入和农民人均纯收入均增长10%左右，城镇居民登记失业率控制在4.5%以内，居民消费价格涨幅控制在4%以下，人口自然增长率控制在6.5‰以内。

实现上述目标，必须要把握好六个方面的着力点：一是必须把项目带动作为扩大需求的根本动力。我市经济发展的外向度较低，消费需求增长缓慢，经济增长在短期内主要依靠投资拉动。要积极谋划和推进一批产业转型、基础设施、重大民生项目，在扩大投资中壮大经济实力、增强发展后劲。二是必须把企业转型升级作为保增长的有力抓手。开发新产品、拉长产业链条、提高企业创新能力，是我市传统优势产业面临的战略选择。要引导和支持企业以市场为导向，积极调整产品结构，加快技术创新、产品创新和管理创新，推动产业转型升级，提升核心竞争力。三是必须把破解瓶颈制约作为助企解困的重要任务。资金、土地、能源供应紧张，要素价格不断攀升，是当前制约企业发展的重要因素。要把服务企业作为当前工作的重中之重，主动为企业协调解决资金、市场等方面的问题，帮助企业纾压解困、渡过难关。四是必须把扩大开放作为加快发展的主要途径。周边地区快速发展的现实启示我们，仅仅依靠现有的产业基础和自身扩张，不可能在新一轮的竞争中抢占发展先机。必须进一步扩大对外开放，加大招商引资力度，积极承接产业转移，吸引资金、技术、人才等要素向我市集聚，才能借力实现跨越式发展。五是必须把城镇化引领作为推动“三化”协调发展的关键举措。城镇化是“三化”协调发展的龙头。洛阳要在中原经济区建设中当示范、作标兵，就必须以新型城镇化为引领，深入开展城乡建设提升活动，加快城乡一体化步伐，实现以产促城、以城带乡、产城融合。六是必须把改善民生作为一切工作的出发点和落脚点。解决好群众的住房难、看病难、上学难、就业难等问题，是各级政府的中心任务。必须坚持以民生为本，不断加大民生投入，办好涉及民生的大事要事，稳步提高人民群众的幸福指数。

三、2012年重点工作

坚持把稳增长、调结构、抓改革、惠民生、促和谐作为政府工作的主线，持续和提升“六加一”攻坚战，确保全市经济社会平稳较快发展，确保人民群众生活水平明显提高。

（一）加快工业结构调整，努力打造特色明显的新型工业化城市。坚持把项目建设作为应对当前复杂局面、培育竞争优势的重要抓手，改造提升传统优势产业，大力发展战略性新兴产业，推动产业总量扩大、结构优化、水平提升。做大做强优势产业。加大技术突破和改造升级力度，引导企业通过技术创新实现制造工艺和产品高端化。以龙头带动、基地支撑、高端突破为着力点，通过招商引资、整合重组，进一步做大做强装备制造、石油化工、能源电力等支柱产业。开工建设725所特种材料装备基地、洛阳石化45万吨PX、一拖集团新型轮式拖拉机核心能力提升等项目，加快推进万基控股集团60万吨铝板带箔等在建项目，做好洛阳石化1800万吨炼油扩能、河南煤化集团100万吨PTA、大鱼沟抽水蓄能电站等项目前期工作，争取中信重工超深矿井提升设备、南车集团城轨车辆组装等项目尽快竣工投产。抓好国机重工产业园等十大园区建设。加快推进洛玻、白马、洛耐、洛单等企业搬迁改造。培育壮大新兴产业。加强新材料、新能源、新能源汽车、生物制药等领域的关键技术研发，加快培育骨干企业，提高产业发展水平。突出抓好中硅高科光伏产业园、普莱柯生物动物疫苗产业化等十大示范项目。加快推进中航锂电产业园等在建项目，尽快开工金诺公司蓝宝石单晶炉及蓝宝石生长新工艺产业化等项目，确保725所钛泵阀基地等项目竣工投产。提升产业集聚水平。加快产业集聚区生产生活配套设施建设，确保所有产业集聚区发展区基础设施全覆盖，公共生活服务平台和供水、供电、供热、供气、污水处理等设施全部建成投用。强化主导产业集群培育，综合运用项目准入、要素配置等手段，推动同类项目、关联项目向园区集中。确保17家产业集聚区新开工亿元以上工业项目150个、竣工70个以上，形成营业收入超百亿元的产业集群12个，营业收入比上年增长35%，固定资产投资力争增长40%以上。增强自主创新能力。加快研发平台建设，新建市级以上工程技术研究中心10个、省级重点实验室1－2家、院士工作站3个。完善区域创新体系，加快产学研军融合发展，抓好新区科技城、大学科技园等创新载体建设，做大做强轴研科技、北玻公司等高新技术企业，支持20家科技型中小企业科技创新。大力实施知识产权推进战略，申报发明专利1300件以上。提升科研成果产业化水平，全年高新技术产业增加值突破200亿元。加快推进节能降耗。坚持强度和总量双控制，完善考核奖惩机制，把好项目准入关，大力淘汰落后产能，确保全年单位生产总值能耗下降3.5%。积极推行合同能源管理，培育节能服务市场体系。抓好重点领域、重点区域、重点行业的节能降耗。加快循环经济试点市建设，做好伊电集团、万基控股、骏马化工等企业及洛宁、嵩县等5个产业集聚区循环经济试点工作。

（二）加强“三农”工作，努力打造现代化新型农村。认真落实各项惠农政策，加强农业基础设施建设，加快农业现代化进程，促进农业增效、农民增收和农村发展。继续做好“三篇文章”。加快农业结构调整，新发展蔬菜、花卉苗木、林果、牡丹27万亩，新建、改造标准化畜禽养殖场（区）10个，新增休闲农业园区50个，完成土地流转75万亩以上。提高农业科技服务水平。加大科技投入，完善农业科研、技术推广和人才队伍体系，大力推广先进适用技术，强化农业科技支撑，不断提高农产品市场竞争力。新增农机动力5万千瓦，推广新型农机具1000台，进一步提高农业机械化程度。加强农产品质量监管，确保产品质量安全。提升农业产业化水平。重点扶持一批农副产品精深加工、冷链物流等龙头企业，新发展产业化龙头企业10家、专业合作社100家。提高农业综合生产能力。严格落实耕地保护制度，确保粮食总产稳定在40亿斤以上。完成水库除险加固50座，加快农田水利设施建设，不断增加有效灌溉面积。争取小浪底南岸灌区、伊洛河治理等项目尽快立项。改善农村人居环境。加强农村水、电、路、气等基础设施建设，硬化通组通户道路 2000公里 。新建户用沼气1万座、服务网点100个。解决农村安全饮水20万人。

（三）加快服务业发展，努力打造现代服务业高地。坚持把服务业发展作为扩大内需、产业转型、改善民生的综合性举措，进一步做大总量，提升水平。大力发展信息产业。重点支持呼叫中心、数据处理、物联网、电子商务、软件外包等产业发展，积极培育各具特色、互为补充的信息产业园区。突出抓好中移动（洛阳）呼叫中心、炎黄科技园、国际科技创新产业园等十大信息服务业项目。大力发展现代物流业。加快区域性物流枢纽建设，重点发展工业品、农产品、建材、汽车摩托配件及冷链物流。突出抓好国龙物流园区、宏进农副产品国际物流中心、格力电器物流配送中心等十大物流项目，确保东汉禽业冷库及物流配送中心、新春都冷链物流中心、一拖物流园区等项目年内竣工投用。大力发展现代金融业。积极吸引境内外金融机构在洛设立区域性总部、功能中心、后台服务中心和分支机构，完善金融市场体系，不断创新金融产品。建立中小企业信用评级和贷款风险补贴机制。规范整顿担保机构，优化金融生态环境，防范化解金融风险。大力发展现代商贸业。重点抓好泉舜财富购物中心、建业凯旋广场等20个大型商贸项目。深入推进“万村千乡市场工程”，建成乡镇连锁商业中心和乡村直营店30家以上。鼓励丹尼斯百货等商贸流通企业实施“农超对接”，加快形成现代农产品流通网络体系。做大做强特色餐饮业，打造洛阳餐饮品牌。大力发展社区服务业。积极开展家政服务及社区休闲娱乐服务，培育一批运作规范、连锁经营的龙头企业及养老、医药、保健、代理服务等新兴服务网点。建成放心早餐网点、蔬菜直销网点50个。加强市场指导和调控，促进房地产业平稳健康发展。

（四）推动文化大发展大繁荣，努力打造国际文化旅游名城。坚持文化与旅游融合发展，文物保护与开发利用统筹兼顾，文化事业与文化产业相得益彰，做好传承和创新两篇文章，加快建设国际文化旅游名城和最佳旅游目的地。积极打造河洛文化传承弘扬品牌。依托文化资源优势，突出项目带动，积极推进龙门文化旅游园区、白马寺佛教文化园区、关圣文化产业园区、老城历史文化街区、涧西工业文化遗产街区等十大传统文化展示及提升项目，加快大遗址保护和博物馆展示工程建设，做好丝绸之路、大运河申报世界文化遗产工作。加强河洛文化研究和典籍整理出版工作，筹建河洛文化大型数据库。发展壮大文化旅游产业。高标准办好第30届中国洛阳牡丹文化节等重大节会。实施小浪底西霞院、伏牛山世界地质公园等十大旅游景区提升行动，加大宣传推介力度，完善旅游设施，丰富旅游产品，优化旅游环境，提升服务质量，满足游客多层次需求，力争全年接待游客增加1000万人次。加快发展特色文化产业，提高牡丹画、唐三彩、澄泥砚、青铜器等特色文化产品档次。精心打造3D动漫电影《牡丹》、红太阳演艺剧场等演艺项目。繁荣发展文化事业。完善公共文化服务体系，新建市科技馆，积极推进市群艺馆以及县级图书馆、文化馆、乡镇文化站、农家书屋建设。实施农村数字电影放映工程，确保每村每月放映一场公益电影。扎实开展公益性演出等文化惠民活动，不断丰富群众文化生活。

（五）深化改革开放，努力打造充满活力的开放城市。大力实施开放战略，建立举市开放体制，以改革破解发展难题，以开放促改革促发展。全面深化企事业单位改革改制。已完成改革任务的单位，要进一步理顺产权关系、完善法人治理结构；存在遗留问题的单位，要一企（事）一策，加快推进。全面完成县（市、区）企事业单位改革任务。加快厂办大集体改革。深化文化、旅游、市政、园林、教育等系统改革。进一步创新投融资机制。加快投融资平台整合，完成洛矿集团发行中期票据、企业债券工作，扩大信托、票据、租赁、债券等融资规模。积极推动银企合作，有效解决企业尤其是中小企业融资难问题。设立产业基金、创业投资基金等，引导更多的资金投入实体经济。鼓励中小企业通过风险投资、资金租赁等方式直接融资。推动洛阳银行尽快进入上市程序，争取中信重工、洛阳钼业、普莱柯生物通过证监会审核，全年新增上市企业3－5家。继续深化大招商活动。优化招商引资考核指标，完善招商引资工作机制。紧盯世界500强、中国500强、行业100强企业，瞄准珠三角、长三角、闽东南等重点区域，集群式承接产业转移，引进一批具有龙头带动作用的大项目和品牌项目。积极参加河南省第七届投资贸易洽谈会及第三届产业转移系列对接活动，组织好牡丹文化节投资贸易洽谈会等重大招商活动。不断提升对外贸易水平。积极推进洛阳出口加工区申报工作，争取洛阳经济开发区升格成功。以产业集聚区、加工贸易承接地为平台，在装备制造、太阳能光伏、钢制办公家具等领域，培育一批竞争优势明显、带动能力强的出口产品基地和产业集群。加大政策支持力度，扶持35家出口企业，培育5家进出口总额超1亿美元的企业。发展壮大民营经济。建立各具特色的民营企业产业联盟，增强民营企业整体竞争力。加快中小企业服务体系建设，大力培育骨干企业，重点选择50户“小巨人”企业，在专项资金、人才引进、市场融资等方面予以支持，确保民营经济占生产总值的比重达52%以上。

（六）加快提升中心城区功能，努力打造区域副中心城市。强化经营城市理念，进一步完善功能、提升水平，加快建设“文化为魂、水系为韵、牡丹为媒、产城融合、组团发展、生态宜居”的山水洛阳。提升城市规划品位。围绕“一中心、三板块、五组团、四支撑”的城市总体布局，全面提高城市规划设计水平，彰显历史文化名城特色。抓紧编制中心城市组团发展战略规划、城市分区规划、近期建设规划及专项规划，年底前实现洛北城区控制性详规全覆盖。重点区域的城市设计、建筑色彩和建筑造型必须进行专家评审、社会公示。高标准加快新区建设。重点抓好希望路跨伊河大桥等55项续建工程，开工建设中原大道跨伊河大桥等34项市政工程。加快龙门大道和开元大道沿线、行政副中心、科技城、动力谷等重点区域规划建设。持续推进城区改造。重点在完善路网结构、改善居住环境上下功夫，开工建设城市东环、西环、南环及滨河北路三期等17项市政工程，加快推进唐宫西路、丽春东路等20项续建工程，做好城市轨道交通、北环路等项目前期工作。充分利用地上地下空间，规划建设一批公共停车场。坚持依法拆迁、阳光拆迁、和谐拆迁原则，大力推进城中村、城郊村和旧城改造。完善城市服务功能。加快城市基础设施建设，推进管道天然气向县域、产业聚集区和工业园区延伸，完成洛新线、洛宜线、东环线等建设任务。新增供热面积160万平方米。完成7000户城市居民生活区水表改造。新增公交车300辆、开通加密公交线路20条。提升城市精细化管理水平。按照为民、持续、统筹、同心、务实的要求，巩固提升全国文明城市创建成果。完善城市管理长效机制，建成数字化城市管理系统，重点解决好占道经营、私搭乱建等影响城市形象的问题。深化“畅通洛阳”活动，缓解城市交通拥堵问题。大力实施碧水蓝天工程。确保新区污水处理厂及关林、安乐、新街等三大片区污水管网建成投用，伊滨区污水处理厂开工建设，市区所有污水管网道路完成雨污分流改造，全市污水处理厂正常运行率达90%以上。加快引黄入洛工程和伊河、洛河治理提升进度，积极实施中州渠、伊东渠提升改造和新区轴带水系项目，抓紧治理邙山渠、秦岭防洪渠、涧河和瀍河城区段。加强扬尘控制和机动车尾气治理，确保城区空气质量优良天数达310天以上。完成造林46.9万亩，新增城市绿地100万平方米。

（七）加强城乡互动对接，努力打造城乡一体化示范区。坚持以新型城镇化为引领，搞好城乡统筹和市县联动，不断增强以城带乡、城乡融合发展的能力。加快建设区域综合交通网络。以高速公路、快速通道为支撑，打通对外大通道，完善对内大循环，形成快捷畅通的综合交通体系。加快推进连霍高速洛阳至三门峡段、武西高速洛阳段、310国道洛阳境改建、洛吉快速通道等项目建设；确保龙门大道、310国道白马寺段货车通道改线、机场路大修等工程牡丹文化节前建成通车，洛伊快速通道6月底前建成通车，洛栾高速、郑卢高速洛阳段、小浪底专用线改造等项目年底前建成通车，形成市区到偃师、孟津、新安、伊川、宜阳半小时交通圈，到洛宁、汝阳、嵩县、栾川一小时交通圈，年底前实现县县通高速。加快洛阳机场改扩建工程，确保今年新开和加密航线6－8条，旅客吞吐量达到60万人次以上。注重提升县城和小城镇内涵。要突出特色，提高品位，强化产业支撑，改善居住环境，不断提升县城和小城镇的综合承载能力。加快县（市）供气、供暖、污水和垃圾处理、信息网络等基础设施建设，实现中心城市与县（市）之间、县与县之间互联互通。加快新型农村社区建设。科学编制新型农村社区规划，在城市近郊、产业集聚区、旅游景区、搬迁扶贫新村、移民新村建设新型农村社区试点。研究出台土地、信贷等优惠政策，鼓励投融资公司、房地产开发企业、经济强村集中建设新型农村社区，特别是洛阳新区要发挥示范和引领作用。全年开工建设大型社区50个，新建农村社区服务中心100个。建立完善就业、社会保险、社会救助、医疗卫生、文化、教育、体育等农村公共服务体系，推动城市公共服务向农村延伸、向新型农村社区延伸。以“乡改办、村改居”为抓手，加强新型农村社区管理，提高群众自治水平。放宽城镇户籍限制。完善城镇入户机制，切实解决农民进城就业、住房、社会保障、子女入学等问题，让稳定就业的进城务工人员享受城镇居民同等待遇。积极争取城镇建设用地增加规模与吸纳农村人口进入城市定居规模挂钩试点，吸引更多的农村人口进入城镇。

（八）高度重视民生，努力打造人民满意的和谐城市。今年全市各级财政新增财力主要用于民生项目建设。办好省定、市定福民实事，下大力气解决好群众就业、上学、看病、住房等问题，把更多的公共资源用于扶助弱势群体和困难群众。积极促进全民创业。完善支持自主创业和就业的政策体系，多渠道增加就业岗位，全市新增城镇就业10万人，再就业4万人。发放小额担保贷款12亿元以上，培育发展微型企业700家以上，支持1.5万人创业。加快发展教育、卫生等社会事业。加大教育投入，优化资源布局，促进义务教育均衡发展。全市新建中小学校43所、幼儿园223所，改扩建中小学23所。加强薄弱学校建设，逐步解决“大班额”等突出问题。大力发展职业教育，加快普及高中阶段教育。坚持“一保三放开”，推动多种形式办学。推进基层医疗卫生机构综合改革和公立医院改革，在全市村卫生室实施基本药物制度，年底前实现县域公立医疗机构药物零差率销售全覆盖。开工建设9县（市）卫生监督中心和120急救体系项目。广泛开展全民健身活动，提升竞技体育发展水平。做好人口和计划生育工作，稳定低生育水平。大力发展老龄事业和残疾人事业。努力提高社会保障水平。大力推进全国城乡居民社会养老保险示范县建设。提高城镇居民基本医疗保险报销比例和最高支付限额，加快推行基本医疗和大病救助同步即时结算。新型农村合作医疗报销年封顶线提高至15万元。多策并举保障市场供应，大力整顿市场经济秩序，保持物价总体平稳。健全最低工资标准、职工工资、退休人员基本养老金、社会救助、城乡低保及农村“五保”供养标准与物价联动挂钩机制，合理增加城乡居民特别是低收入群体的收入。完善机关事业单位工资和津补贴制度。加大保障性住房建设力度，全年开工公共租赁房、廉租房、经济适用房、棚户区改造房2.48万套，竣工1.05万套。扎实推进利用住房公积金贷款支持保障性住房建设试点工作。做好灾区倒房重建工作。实施60所农村敬老院提升改造工程。加大扶贫开发力度，完成50个贫困村的整村推进和3000户1.25万人的搬迁扶贫任务，实现扶贫开发和农村低保制度有效衔接。全面创新社会管理。大力实施安全环境创优行动计划，深化煤炭、非煤矿山、危险化学品、烟花爆竹、校车等重点领域、重点行业专项整治，加大隐患排查治理力度，杜绝重特大事故发生。强化食品药品监管，确保人民群众食品用药安全。严格落实信访工作责任制，扎实开展重大决策项目信访风险评估，妥善解决好群众合理诉求。发挥宗教界的积极作用，促进民族团结进步。加强社会治安综合治理，深入开展打黑除恶扫痞等专项行动，探索实施城市区“网格化”管理模式，不断提升人民群众的安全感。加强应急保障体系建设，提高应对突发事件的能力。提升110城市应急联动服务水平，实现各县（市、区）联动服务全覆盖，确保群众诉求办结率和满意率达95%以上。扎实开展双拥工作。做好军转干部、退役士兵安置和优抚对象抚恤优待工作，支持驻洛部队、武警部队、民兵预备役和人民防空建设，促进军民融合发展。同时，积极支持工会、共青团、妇联等人民团体依照各自章程开展工作，发挥好桥梁纽带作用。

总之，民生连着民心。人民群众的幸福，就是我们各级政府的最大追求。我们一定要加倍努力，毫不懈怠，真正把民生工程办成群众满意的民心工程，努力让洛阳人民生活得更加舒心、更加安心、更加顺心。

四、关于政府自身建设

2012年是市委、市政府确定的作风转变年。各级各部门要切实转变工作作风，不断提高行政效能，努力提升服务质量，加快建设全国一流、全省最优的发展环境。

坚持依法行政。严格执行人大及其常委会的决议和决定，依法接受其法律监督和工作监督，自觉接受政协的民主监督。认真办理人大代表议案、建议和政协委员提案。坚持开门立法，建立公民有序参与立法的长效机制，提高政府立法质量和水平。深入推进法治城市创建工作。加强执法队伍建设，规范行政执法行为。加强行政复议工作，健全行政监督体系，认真查处群众反映强烈的突出问题。

提高行政效能。继续深化行政审批制度改革，优化审批工作流程。各级政府和部门要再减少些程序，再缩短些时限，再取消些审批项目，再下放些审批权限，努力形成零障碍、低成本、高效率的投资环境。对于基层、企业和群众反映的问题，必须立即办、主动办、认真办、跟踪办。大力推行“一线工作法”，认真落实首问负责、限时办结、服务承诺、全程代理等制度。加强电子政务建设，全面推行网上审批，为企业、基层和群众提供更加便捷、更加高效的服务。

强化科学运作。遵循和运用好市场经济规律、群众工作规律、具体问题具体分析规律，努力破解各种发展障碍和瓶颈制约。坚持“三重一大”事项集体决策，积极开展网络问政工作，健全重大事项公众参与、社会听证、专家论证、咨询评估等机制，提高政府民主决策水平和科学运作能力。要加强岗位培训，提高公务员素质，做到在复杂疑难问题面前有破解招数，在重大突发事件面前有应对能力。深入开展争创“人民满意公务员集体”和“人民满意公务员”活动，努力打造一支政治坚定、业务精湛、作风过硬、人民满意的公务员队伍。

锤炼务实作风。大力弘扬“包容开放、创业创新、实干争先、负重奋进”的洛阳精神，进一步凝聚发展合力。始终把群众的事情、企业的事情当作大事、要事来办，全心全力为企业和群众办实事、解难题。大力精简会议、文件，转变会风、文风，不说空话、套话，把更多的精力放在抓落实、促发展上。完善重大项目、重点工作跟踪落实机制，做到有令必行、有禁必止，确保政府的每一项工作都有人执行落实，违反制度有人承担责任，切实提高政府的执行力和公信力。加强政府信用体系建设，完善失信惩戒、责任追究等工作机制。严格落实对企业和投资者的优惠政策，切实兑现对企业、对人民群众的各项承诺。

严格廉洁从政。认真落实党风廉政建设责任制，完善惩治和预防腐败体系，强化对权力运行的制约和监督。严格财政经费、政府投资项目管理，全面落实“三公”经费公开制度，完善政府投资项目监督管理办法，杜绝劳民伤财的形象工程、“政绩工程”，反对讲排场、比阔气。加大政务公开力度，让政府的每一项工作都置于人民的监督之下。有效防范和坚决查处公务人员利用职务之便，干预和插手工程招投标、土地招拍挂、政府采购、国有产权和矿权交易等行为。加大行政问责和行政监察力度，对行政不作为、乱作为现象要专项治理，对各类违法违纪案件要一查到底，坚决惩处。下大力气纠正各种损害群众利益的不正之风，以为民、务实、清廉的政府形象，赢得人民群众的信任和支持，让政府公簿的每一页都写满公开、公平和正义。

各位代表！宏伟的蓝图引领着我们，庄严的使命激励着我们，美好的未来召唤着我们。让我们高举中国特色社会主义伟大旗帜，认真落实科学发展观，坚持“四个重在”的实践要领，在省委、省政府和市委的坚强领导下，凝心聚力、开拓进取，奋力谱写洛阳跨越式发展的新篇章，为早日实现福民强市目标、全面走在中原经济区建设前列而努力奋斗！
